# Chinese and Australians showed difference in mental time travel in emotion and content but not specificity

**DOI:** 10.3389/fpsyg.2015.00879

**Published:** 2015-06-26

**Authors:** Xing-Jie Chen, Lu-Lu Liu, Ji-Fang Cui, Ya Wang, David H. K. Shum, Raymond C. K. Chan

**Affiliations:** ^1^Neuropsychology and Applied Cognitive Neuroscience Laboratory, Key Laboratory of Mental Health, Institute of Psychology, Chinese Academy of SciencesBeijing, China; ^2^University of Chinese Academy of SciencesBeijing, China; ^3^Information Center, National Institute of Education SciencesBeijing, China; ^4^Behavioural Basis of Health Program and Menzies Health Institute Queensland, Griffith UniversityGold Coast, QLD, Australia

**Keywords:** cultural differences, future thinking, mental time travel, autobiographical memory, Chinese, Australian

## Abstract

Mental time travel refers to the ability to recall episodic past and imagine future events. The present study aimed to investigate cultural differences in mental time travel between Chinese and Australian university students. A total of 231 students (108 Chinese and 123 Australians) participated in the study. Their mental time travel abilities were measured by the Sentence Completion for Events from the Past Test (SCEPT) and the Sentence Completion for Events in the Future Test (SCEFT). Results showed that there were no cultural differences in the number of specific events generated for the past or future. Significant differences between the Chinese and Australian participants were found mainly in the emotional valence and content of the events generated. Both Chinese and Australian participants generated more specific positive events compared to negative events when thinking about the future and Chinese participants were more positive about their past than Australian participants when recalling specific events. For content, Chinese participants recalled more events about their interpersonal relationships, while Australian participants imagined more about personal future achievements. These findings shed some lights on cultural differences in episodic past and future thinking.

## Introduction

Mental time travel refers to the ability to mentally travel through time, by recalling one’s past events and envisioning possible future events (Arzy et al., 2008; Botzung et al., 2008). Recalling the past and imagining the future shared a similar cognitive process and memory system (Addis et al., 2007; Schacter and Addis, 2007a,b; Schacter et al., 2012). Studies have shown that how a person represents past information is influenced by cultural myths and social narratives, and it is also related to the self in different cultural contexts (Nelson, 2003).

Previous studies mainly focused on cultural differences in episodic specificity of one’s autobiographical memory. And many of them defined specificity as the details contained in the description of events. Wang (2009) reviewed previous studies and found that compared with people from western cultures, Asians or Asian Americans usually generated less specific details.

Regarding the dimension of time, according to the episodic simulation hypothesis, past experiences are the constructive scripts when people imagine about the future (Schacter and Addis, 2007a,b; Schacter et al., 2008). Thus cultural differences in memory about the past may also be reflected in thinking about the future. To date, there is only one study exploring cultural differences in specificity of mental time travel (Wang et al., 2011). In the study, Chinese and European American participants were asked to free recall and imagine specific personal events within/after a week, a year or 10–15 years. The number of episodic details included in the free recall responses was then assessed (Wang et al., 2011). Results of the study suggested that Chinese participants generated less specific information than their European American counterpart both in recalling the past and imagining the future. However, Wang et al. (2011) did not explore another indicator of specificity, namely, whether participants generate events that happened/will happen in a specific place and time that last no more than one day (Wang, 2009) in the study.

Emotional valence of the events people generated about the past and the future is also important for mental time travel. There is a positivity bias in mental time travel, that is, people would generate more positive events than negative events (Walker et al., 2003b; Killingsworth and Gilbert, 2010). This bias has been found to be stronger when thinking about the future (Berntsen and Bohn, 2010). This is because for the unknown future, there are more uncorrected positive illusions than in the past (Cacioppo and Gardner, 1999). Moreover, future thinking is usually related to future plans and personal goals and people prefer to think more positively about these. Whether the degree of positivity bias is different between cultures is an open question.

To date, studies investigating cultural differences about the emotional valence in mental time travel are very limited. Some studies about past memory showed that in eastern cultures, people would like to see the two sides of an event (dialecticism), that is, viewing the positive aspect of a negative event, and vice versa (Spencer-Rodgers et al., 2010; Miyamoto et al., 2014). In western culture, however, negative emotional experiences can be seen as a kind of loss of self-control so that they view negative emotions as undesirable (Eid and Diener, 2001). Moreover, European Americans have been found to prefer to discount the past while Chinese have been found to respect the past more when they were asked to rate their current subjective well-being (Kim et al., 2012). Therefore, people in eastern cultures like Chinese may recall more positive events.

Shao et al. (2010) explored the emotional valence of mental time travel in European Americans and Chinese, and found positivity bias in both Chinese and European Americans, but European Americans were far more positive than Chinese for both past and future events. However, people in different cultures may display a different pattern of results depending on the specificity of events recalled. Therefore, in the present study, we aimed to examine cultural differences on emotional valence in mental time travel, particularly for specific events.

Apart from studying specificity and emotional valence of past and future thinking, researchers have recently started to pay attention to the content of the events people generated. Self concept has been found to be associated with the content of imagined events (Shao et al., 2010). Cross-cultural research has shown that people from eastern cultures tended to emphasize social harmony and focus more on interpersonal relationships, while people from western cultures tended to emphasize individuality and pay more attention to individual achievements. Such cultural differences were reflected in the content of autobiographical memory recall in that Chinese have been found to focus on the social environment and relationships using more other-references in autobiographical narratives than Americans (Han et al., 1998; Ji et al., 2000).

In summary, mental time travel, including episodic past and future thinking, may be affected by cultural and social contexts. Cultural differences may be found at the level of specificity, emotional valence as well as content. The aim of the present study was to investigate cultural differences in these three aspects of mental time travel between Chinese and Australians using the Sentence Completion for Events from the Past Test (SCEPT; see Raes et al., 2007) and the Sentence Completion for Events in the Future Test (SCEFT; Anderson and Dewhurst, 2009). Australians and Chinese were chosen to represent western and eastern cultures based on their individualism versus collectivism profiles. Australians have a relatively high individualism but low collectivism profile, while Chinese have a low individualism but high collectivism profile (Oyserman et al., 2002). These two populations have also been compared as representative of western and eastern cultures in previous studies (e. g. Bain et al., 2011; Shahaeian et al., 2013; Hiew et al., 2015). The sentence completion tests rather the commonly used cue-word tasks were used in this study to measure mental time travel. This is because Raes et al. (2007) suggested that these tests are more sensitive for testing the specificity of episodic memory in non-clinical population and they do not explicitly ask participants to generate specific events. On the other hand, the cue-word tasks explicitly require participants to generate specific events, and they were usually used to compare clinical populations and healthy controls. Healthy controls usually show very good performance in such tests (D’Argembeau et al., 2008; Lind and Bowler, 2010; Brown et al., 2013).

We hypothesized that for both Chinese and Australian participants, they would generate more specific events about the past than that of the future. For cultural differences on specificity, no study has examined the specificity (events with specific place and time and lasted no more than one day) of mental time travel; therefore, we did not derive a specific hypothesis. Regarding emotional valence of specific events, both Chinese and Australians were expected to show positivity bias for future events. As for past events, it was expected that Chinese participants would generate more experiences with positive emotion. As for content of events generated, while Chinese would generate more events related to social context and interpersonal relationships. For Australians, they would generate more events about individuality.

## Materials and Methods

### Participants

A total of 231 college students participated in the study, including 108 Chinese and 123 Australian students (11 Asian students studying in Australia were excluded). The Chinese students (30 males and 78 females; mean age: 20.14 years; SD = 1.42) were from the first or second year cohort at Huazhong Normal University, Wuhan and China Youth University for Political Sciences, Beijing. The Australian students (27 males and 96 females; mean age: 19.22 years; SD = 2.80) were from the first year cohort at Griffith University.

### Materials

#### Sentence Completion for Events from the Past Test (SCEPT; Raes et al., 2007)

The SCEPT has 11 sentence stems for participants to recall past experiences. For example, “When I think back to/of …”. Participants were required to complete the sentence stems in any form, but for each response the contents must be about different topics. The completed sentences were rated in terms of three aspects: (1) specificity: specific (a specific event happening at a particular time and place within a day), extended (a specific event lasting for more than one day), categorical (a general event belonging to a category), semantic associates (semantic information), and omission (participants could not recall anything; Raes et al., 2007; Anderson and Dewhurst, 2009); (2) emotional valence: positive, negative and neutral; (3) content: life-threatening events, exploration/recreation, relationships, achievement/mastery, guilt/shame, drug/alcohol, hospitalization/stigmatization, failure, happy events, career, neutral events, and events not classifiable (Raffard et al., 2010). We chose the coding system based on the following reasons: first, the coding system was used in classifying self-defining memories (Raffard et al., 2010); Second, self is very important in mental time travel (Shao et al., 2010); Third, people’s self has been found to be different across cultures (Shao et al., 2010).

#### Sentence Completion for Events in the Future Test (SCEFT; Anderson and Dewhurst, 2009)

The SCEFT has 11 sentence stems for participants to imagine possible events in the future. For example, “When I look forward to…”. The requirements and ratings for the test were the same as the SCEFT.

### Procedure

The current study was approved by the ethics committee of the Institute of Psychology, Chinese Academy of Sciences and Griffith University. All participants were given a brief written introduction about the study. All of them provided written informed consent before the commencement of the study. The Chinese students filled in the simplified Chinese version of SCEPT and SCEFT; the Australian students filled in the English version. The order of administration of the two tests was counterbalanced across participants. Chinese participants were paid 50 RMB for their participation (for this and some other measures not included in this study). Australian participants were given a one-hour course credit for their participation.

The answers of each sentence were rated by two raters according to the scoring criteria. For items where the raters did not agree, their scores were discussed and reconciled with a third rater. The inter-rater reliabilities (calculated with Cohen’s Kappa) are good (specificity: *K* = 0.81; emotional valence: *K* = 0.85; content: *K* = 0.88).

.

### Results

The proportion of events generated in each category and all aspect of the ratings were the main measures of the participants’ performances (see **Table 1**).

**Table 1:** Mean (SD) proportions of different response categories across tasks in Chinese and Australian participants.

	Chinese		Australian	
	SCEPT	SCEFT	SCEPT	SCEFT
**Specificity**				
Specific events	0.37 (0.18)	0.22 (0.13)	0.33 (0.17)	0.22 (0.16)
Extended events	0.23 (0.14)	0.22 (0.16)	0.23 (0.12)	0.26 (0.15)
Categoric events	0.20 (0.13)	0.38 (0.19)	0.15 (0.10)	0.24 (0.16)
Semantic associates	0.20 (0.14)	0.18 (0.15)	0.27 (0.15)	0.26 (0.15)
omission	0.003 (0.02)	0.002 (0.02)	0.01 (0.05)	0.009 (0.03)
**Emotion**				
Positive	0.29 (0.16)	0.42 (0.18)	0.22 (0.14)	0.40 (0.15)
Neutral	0.51 (0.18)	0.55 (0.17)	0.57 (0.17)	0.56 (0.15)
Negative	0.19 (0.13)	0.03 (0.06)	0.20 (0.14)	0.03 (0.06)

*SCEPF: Sentence Completion for Events from the Past Test; SCEFT: Sentence Completion for Events from the Future Test*.

#### Specificity

The correlation between the percentage of specific past experiences and the percentage of specific future imagination was calculated (Chinese: *r* = 0.320, *p* = 0.001; Australians: *r* = 0.207, *p* = 0.022), indicating a significant relationship between past and future thinking.

For each category of specificity (specific, extended, categorical, and semantic associates), a 2 (Group: Chinese, Australians) × 2 (Time Orientation: past, future) ANOVA was conducted. The results showed that for specific events, the main effect of Group was not significant [*F*(1,229) = 0.83, MSE = 0.032, *p* = 0.364, η_*p*_^2^ = 0.004), indicating no significant difference between Chinese and Australian participants. The interaction of Time orientation and Group was also not significant [*F*(1,229) = 2.22, MSE = 0.019, η_*p*_^2^ = 0.010]. However, the main effect of Time orientation was significant in that participants generated more specific past events (*M* = 0.35) than future events (*M* = 0.22) [*F*(1,229) = 105.18, MSE = 0.019, *p* < 0.001, η_*p*_^2^ = 0.315].

For extended events, the main effects of Time Orientation [*F*(1,229) = 0.86, MSE = 0.017, η_*p*_^2^ = 0.004] and Group [*F*(1,229) = 2.96, MSE = 0.024, η_*p*_^2^ = 0.013] and the interaction effect between Time Orientation and Group were not significant [*F*(1,229) = 2.62, MSE = 0.017, η_*p*_^2^ = 0.011]. For categorical events, the main effects of Time Orientation [*F*(1,229) = 149.87, MSE = 0.015, *p* < 0.001, η_*p*_^2^ = 0.396] and Group [*F*(1,229) = 33.34, MSE = 0.030, *p* < 0.001, η_*p*_^2^ = 0.127], and the interaction effect [*F*(1,229) = 17.11, MSE = 0.015, *p* < 0.001, η_*p*_^2^ = 0.07] were all significant. Results of simple main analyses suggested that in both recalling past and imagining future conditions, the Chinese participants generated more categorical events than the Australian participants [past: *F*(1,229) = 9.13, MSE = 0.010, *p* = 0.003, *d* = 3.277; future: *F*(1,229) = 36.37, MSE = 0.030, *p* < 0.001, *d* = 5.89]. And the group difference was much larger in the future condition. For semantic associates, only the main effect of Group was significant [*F*(1,229) = 24.41, MSE = 0.015, *p* < 0.001, η_*p*_^2^ = 0.096], indicating that Australian participants (*M* = 0.27) generated more semantic associates than Chinese (*M* = 0.19).

#### Emotion

**FIGURE 1:**
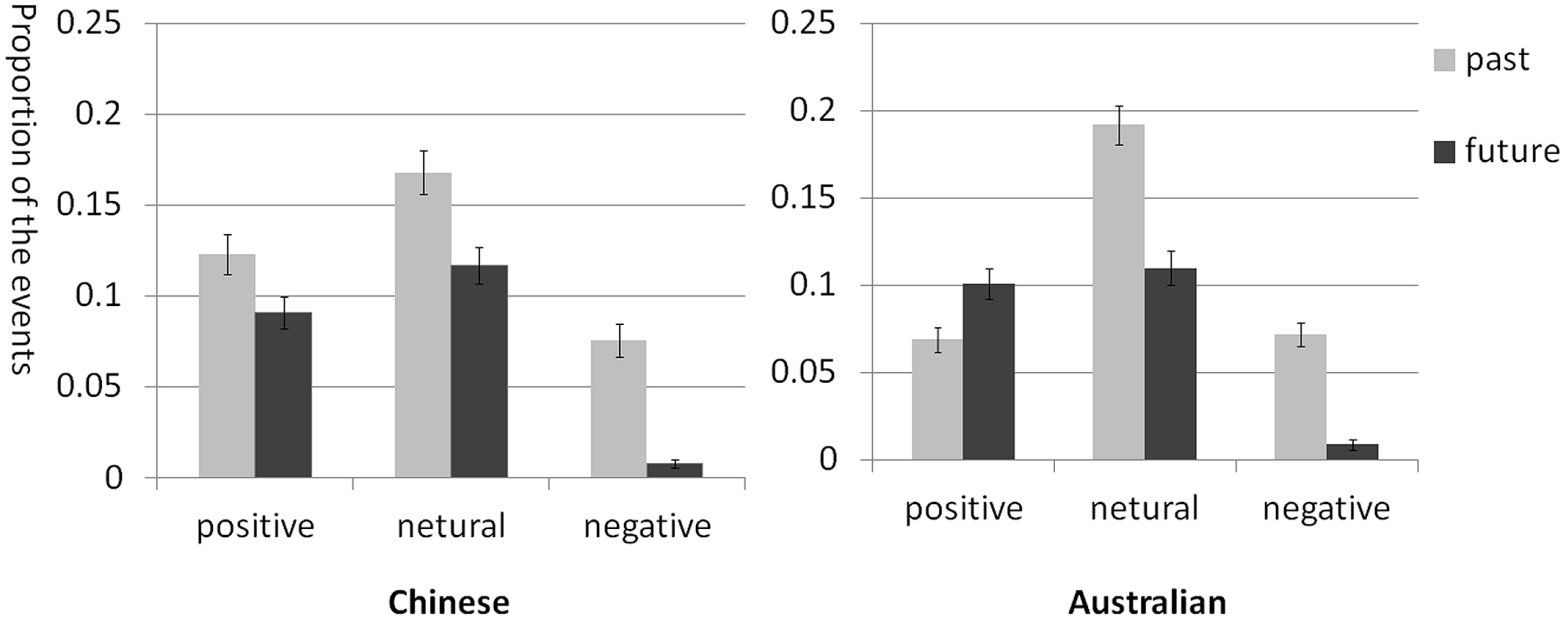
**Mean proportions of specific events with different emotional valences in Chinese and Australians**.

Because of our interest in specific events, we conducted a 2 (Group: Chinese, Australian) × 2 (Time Orientation: past, future) × 3 (Emotional Valence: positive, neutral, negative) mixed ANOVA to analyze the effect of emotional valence on specific events. Results showed that the main effects of Time Orientation [*F*(1,229) = 105.18, MSE = 0.006, *p* < 0.001, η_*p*_^2^ = 0.315] and Emotion [*F*(1,229) = 95.26, MSE = 0.007, *p* < 0.001, η_*p*_^2^ = 0.294] were significant. The participants recalled more specific events (*M* = 0.117) than imagined future specific events (*M* = 0.073). And they generated more neutral events (*M* = 0.147) than positive events (*M* = 0.096), and more positive events than negative events (*M* = 0.041). The main effect of Group was not significant [*F*(1,229) = 0.83, MSE = 0.011, *p* = 0.364, η_*p*_^2^ = 0.004]. The interaction between Time Orientation and Emotion was significant [*F*(2,458) = 21.06, MSE = 0.008, *p* < 0.001, η_*p*_^2^ = 0.084], while the interaction between Time Orientation and Group [*F*(1,229) = 2.22, MSE = 0.006, *p* = 0.138, η_*p*_^2^ = 0.010], and the interaction between Emotion and Group [*F*(1,229) = 3.46, MSE = 0.007, *p* = 0.064, η_*p*_^2^ = 0.015] were not significant. There was a significant three-way interaction [*F*(1,229) = 8.03, MSE = 0.006, *p* = 0.005, η_*p*_^2^ = 0.035]. Further analysis showed that for both Australians and Chinese, they recalled more neutral (*p*s < 0.001, *d*_Chinese_ = 4.62, *d*_Australian_ = 7.80) and negative (*p*s < 0.001, *d*_Chinese_ = 11.26, *d*_Australian_ = 10.43) past events than imagining future events. However, for positive events, Chinese recalled more positive past events than imagined future events (*p* < 0.001, *d* = 3.56), while Australians imagined more positive events than recalled past events (*p* < 0.001, *d* = –3.56) (**Figure 1**). Group comparison showed that when thinking about the past, Chinese thought about more positive specific events than Australians (*p* < 0.001, *d* = 2.44) but not for the neutral and negative events (both *p*s > 0.05). As for the future, the two groups of participants did not show significant differences in positive, neutral, or negative specific events (all *p*s > 0.05).

#### Content

A 2 (Group: Chinese, Australian) × 2 (Time Orientation: past, future) × 12 (Content: the 12 categories of the events) MANOVA was conducted to examine the effects of Group and Time Orientation on the 12 categories of events (all generated events) (see **Table 2**). Because our main interests were the main effects of Group and Time Orientation, only the results for the main effects of these two factors and the interaction effects involving these factors were presented. Results showed that the main effect of Group [*F*(1,229) = 4.03, MSE = 0.027, *p* = 0.046, η_*p*_^2^ = 0.017), the interaction between Content and Group [*F*(11,229) = 6.63, MSE = 0.009, *p* < 0.001, η_*p*_^2^ = 0.028], the interaction between Time Orientation and Content [*F*(11,229) = 38.61, MSE = 0.006, *p* < 0.001, η_*p*_^2^ = 0.144), and the three-way interaction of Group, Time Orientation, and Content [*F*(11,229) = 5.73, MSE = 0.037, *p* < 0.001, η_*p*_^2^ = 0.024] were significant. *Post hoc* comparisons (with Bonferroni corrections) for the three-way interaction were done based on each content category. Results showed that for life-threatening events, Australian participants imagined more than Chinese participants (*p* = 0.032, *d* = 0.292) while no significant difference was found for recalling this type of event in the past. For events about exploration/recreation, Chinese participants recalled more than the Australians (*p* < 0.001, *d* = 6.945) while no significant difference was found when participants were imagining the future. For events about relationships, Chinese participants recalled more than Australian participants (*p* = 0.003, *d* = 0.391) while no significant difference was found in the future condition. For the events about Drug/alcohol, Australian participants recalled more than Chinese participants (*p* = .029, *d* = 0.310) while no significant difference was found in the future condition. For events about career, Chinese participants imagined more than Australians (*p* = 0.026, *d* = 1.018) while no difference were found for past recall. For events about achievement/mastery, Australian participants imagined more than Chinese (*p* = 0.026, *d* = 0.529) while no difference was found for past recall.

**Table 2:** Mean (SD) proportions of different categories of events generated by Chinese and Australian participants.

	Chinese		Australian	
	SCEPT	SCEFT	SCEPT	SCEFT
Life-threatening	0.05 (0.07)	0.002 (0.02)	0.06 (0.08)	0.009 (0.03)
Exploration/recreation	0.15 (0.11)	0.08 (0.08)	0.08 (0.09)	0.08 (0.08)
Relationships	0.18 (0.13)	0.14 (0.11)	0.13 (0.12)	0.12 (0.10)
Achievement/mastery	0.09 (0.07)	0.08 (0.09)	0.08 (0.08)	0.11 (0.08)
Guilt/shame	0.03 (0.05)	0.003 (0.02)	0.03 (0.05)	0.004 (0.02)
Drug/alcohol	0.0008 (0.009)	0.0008 (0.009)	0.006 (0.02)	0.002 (0.01)
Hospitalization/stigmatization	0.005 (0.02)	0.0008 (.009)	0.002 (0.01)	0
Failure	0.01 (0.04)	0.002 (0.01)	0.007 (0.03)	0.002 (0.01)
Happy events	0.06 (0.07)	0.08 (0.08)	0.07 (0.007)	0.09 (0.09)
Career	0.03 (0.05)	0.13 (0.10)	0.03 (0.06)	0.08 (0.07)
Neutral event	0.22 (0.13)	0.34 (0.13)	0.28 (0.14)	0.34 (0.15)
Not classifiable	0.18 (0.15)	0.13 (0.13)	0.23 (0.15)	0.15 (0.12)
Omission	0.004 (0.02)	0.002 (0.02)	0.01 (0.05)	0.009 (0.03)

## Discussion

The present study explored cultural differences in specificity, emotional valence, and content in mental time travel (past and future) between Chinese and Australians. The main findings are: there was no cultural difference in specificity but cultural differences were found in emotional valence and content. Compared to Australian participants, Chinese participants recalled more specific positive events than Australians. In addition, Chinese participants generated more events about interpersonal relationships, exploration/recreation, and career, while Australian participants generated more events about life-threatening, drug/alcohol, and achievement/mastery.

For specificity, the number of specific events recalled and the number of future events imagined were found to be significantly correlated. In addition, both Chinese and Australian participants generated more specific events about the past than about the future. These results supported the episodic simulation hypothesis that envisioning future events required additional process to recombine the features of past experiences and form into new events although the two processes shared a similar memory system (Schacter et al., 2007). However, our study did not find any cultural differences in specificity. To date, there is only one study that explored cultural differences in mental time travel (Wang et al., 2011). Their results indicated that regardless of recalling the past or imagining the future, people in western culture like European Americans generated more specific information and details than Chinese. This finding was explained in terms of the high-elaborative memory of conversations between mothers and children during childhood. The inconsistent results between the present study and those in Wang et al.’s (2011) study could be due to the different methods used to measure mental time travel and different definition of specificity. Wang et al. (2011) asked participants to describe specific experiences and encoded the number of specific information in the description. In our study, however, we used a different measure which asked participants to complete sentence stems without explicit instruction on specificity. We then rated the specificity of the event (specific events last from minutes to hours within one day) which focused on the characteristics of the events themselves. Because the amount of information contained in a sentence is limited, our method may not be suitable to capture detailed information. However, sentence completion test also has its own advantages. As mentioned in Wang’s (2009) review, cultural differences in episodic specificity of mental time travel may be related with language styles, norms of expression and the test context. The explicit instructions on specificity in previous studies may have some priming effects (which might be different across cultures) on these aspects thus introduce a confounding effect. Sentence completion test, without explicit instruction on specificity, may be more accurate and sensitive to measure mental time travel from spontaneous generated events.

For emotional valence of the specific events, Chinese and Australian participants generated similar positive events for the future, but Chinese participants generated more positive events for the past than Australian participants. This may be due to three reasons. First, it has been well documented that people tend to have a positivity bias when thinking about the past (Walker et al., 2003b; Finnbogadottir and Berntsen, 2013) because they would like to maintain a positive self in the present (Walker et al., 2003b; Walker and Skowronski, 2009). However, compared with East Asians, European Americans often discount their past and focus more on the present and future (Spears et al., 2000). Second, European Americans usually disconnect their past from the present (Briley, 2009; Kim et al., 2012). As a result, when thinking about the past, they would neglect some information such as some emotional experiences associated with the events. East Asians, on the other hand, behave in an opposite way. For example, Chinese people tend to care more about the past especially when considering the present and future and directing their behaviors toward it (Levinson and Peng, 2007; Ji et al., 2009; Guo et al., 2012). Third, in western cultures, negativeemotional experiences are regarded as undesirable and might imply losing self-control (Barr-Zisowitz, 2000; Eid and Diener, 2001). As a result, people in western cultures tend to discount or neglect negative events. However, because of dialecticism, Chinese people would reevaluate the negative events and turn them into comparatively positive ones (Miyamoto et al., 2014).

We also analyzed the content of the recalled or imagined sentences. In the 12 classifications adapted from Raffard et al. (2010), cultural differences were found in life-threatening, exploration/recreation, relationships, achievement/mastery, drug/alcohol, and career. And most of these differences are consistent with the generally accepted cultural differences in the dimension of collectivism and individualism (Triandis et al., 1988). For instance, Chinese participants provided more events about exploration/recreation, relationships. All these things are related to interpersonal interactions with the people around them such as traveling with family and friends (exploration/recreation), and break ups or fall-in-love with girlfriends/boyfriends (relationships). On the other hand, for achievement/mastery, Australians generated more events than Chinese and mainly in the future events which reflect the pursuing of individuality in western cultures (Wang et al., 2011). This is also consistent with the interdependent self in Chinese culture and independent self in western culture (Markus and Kitayama, 1991). However, in the classification of career, Chinese participants generated more than Australians when imagining the future. We propose that this may be an exceptional situation for Chinese college students at the moment because they face a lot of pressure in looking for a job after graduation (Li et al., 2011) and parents’ expectation for them was finding a good job. This is supported by the fact that Chinese participants’ specific responses about career were about looking for jobs. Also, in some other categories like life-threatening, and drug/alcohol, Chinese and Australian participants also displayed differences. Australian participants imagined more events about life-threatening, and recalled more events about drug/alcohol. For the events about life-threatening, the events Australians generated were mainly about death and disease, death is a social taboo in Chinese culture (Shek, 2010), thus Chinese participants may be reluctant to think about it. For the events about drug/alcohol, the number of events generated by both groups of participants is very small (less than 0.5%) compared with other categories, and most of the answers were about alcohol. This suggested that in both cultures, the college students did not have many such experiences. The reason that Australian participants reported comparatively more events may be that alcohol was not allowed in Chinese university. Thus Chinese participants would prefer not to report these events.

There are some limitations in the present study. First, the samples in our study were college students and their lives are relatively simple. This could be a potential factor that influences the sensitivity for measuring cultural differences in the specificity of mental time travel. Further studies should recruit participants with a wider range of background from the community. Second, in our study, only participants from China and Australia were chosen as the representations of eastern and western cultures. Future studies should recruit participants with other culture backgrounds to further explore cultural difference in mental time travel. Third, there are a lot of factors that can affect mental time travel. However, we only focused on mental time travel, particularly only on specificity, emotional valence, and content, and did not take into consideration other characteristics of the participants such as personality which has been reported to closely related to autobiographical memory or future thinking (e.g., Walker et al., 2003a; Barnier et al., 2004; Ritchie et al., 2009). Because personality has been found to differ between cultures, it is difficult to decide whether personality mediates cultural differences on mental time travel. Future studies should explore more factors that influencing mental time travel and the potential interactions between these factors.

In summary, the present study explored cultural differences in mental time travel in terms of specificity, emotion, and content. We did not find any cultural difference in specificity. However, for emotion and content, we found that while Chinese people generated more specific positive events about the past and they tended to think more about things relating to interpersonal relationships, Australians generated more neutral events about the past and they tended to think more about things relating to individual pursuits. Moreover, for the specific events, Chinese and Australian participants generated similar positive events for the future, but Chinese participants generated more positive events for the past than their Australian counterparts.

## Conflict of Interest Statement

The authors declare that the research was conducted in the absence of any commercial or financial relationships that could be construed as a potential conflict of interest.
